# Stereoselective Phytotoxicity of HCH Mediated by Photosynthetic and Antioxidant Defense Systems in *Arabidopsis thaliana*

**DOI:** 10.1371/journal.pone.0051043

**Published:** 2013-01-17

**Authors:** Qiong Zhang, Cong Zhou, Quan Zhang, Haifeng Qian, Weiping Liu, Meirong Zhao

**Affiliations:** 1 College of Biological and Environmental Engineering, Zhejiang University of Technology, Hangzhou, China; 2 Institute of Environmental Science, College of Environmental and Resource Sciences, Zhejiang University, Hangzhou, China; Virginia Tech, United States of America

## Abstract

**Background:**

Hexachlorocyclohexane (HCH) has been used for plant protection and sanitation world-widely, and its isomers have been detected in water, soil, and air as well as in vegetation. As a sink for lipophilic pollutants, vegetation is very important for the degradation and fate of organic contamination; however, little was known about their phytotoxicity and mechanisms of toxic effect. In this study, the stereoselective phototoxicity of four isomers (α, β, γ, and δ) of HCHs mediated by independent as well as interconnecting systems of photosynthesis and enzymatic antioxidant defense system in *Arabidopsis thaliana* were assessed.

**Principal Findings:**

Our results revealed that all the HCHs not only stimulated the activities of catalase (CAT) and peroxidase (POD), but also inhibited the activity of superoxide dismutase (SOD). In photosynthesis system, the photosynthetic efficiency of PSI and PSII were all down regulated. Meanwhile, results from both systems showed that δ-HCH was the most toxic one, while α-HCH the least in *Arabidopsis thaliana*.

**Conclusions:**

For the first time, stereoselective effects of different isomers of HCH in plant were demonstrated. And the results suggest that it requires further research to fully elucidate the environmental toxicity and their mechanisms.

## Introduction

Hexachlorocyclohexane (HCH), a highly recalcitrant organochlorine pesticide (OCP), has been widely used in agriculture, public health, and ectoparasite controls since 1940 s [1]. Technical HCH production between 1945 and 1992 was estimated to be 1400 kt, with approximately 400 kt of that were contributed by US producers [2]. Although HCH usage has been banned for decades, the production of HCH is still allowed in the EU (Stockholm Convention in 2004). Besides, a large amount is still used in developing countries because of its low cost [3]. Therefore, the residues of HCH may still be present in soil and other environmental matrices, and may accumulate in food crops and pose potential health risk to humans [4]. The common characteristic of OCPs is the persistence in the environment and the bioconcentration in organism lipid compartments [5]. For example, HCH residues in a contaminated point-source in German were analyzed by Ricking and Schwarzbauer [6] and they found that groundwater and a riverine sediment core was heavily affected by the HCH and its degradation products in the same industrial point source. Because of the high bioconcentration factor and its longer half-life, HCH had been detected in human milk from different countries [7]. Technical grade HCH is a mixture of eight isomers [α (contains a pair of enantiomers), β, γ, δ, ε, η, θ], the toxic effect caused by HCH isomers is mainly focused on animals, despite that plants are providing vital ecosystem service. For instance, researchers have reported the central nervous system, reproductive and endocrine damage induced by HCH isomers in rats [8]–[10]. Besides, comparing with other HCH isomers, β-isomer may currently be the most toxicologically significant HCH due to the reports of its estrogenic effects in mammalian cells, laboratory mammals, and fish [7]. Growing vegetation can absorb OCPs through their roots or leaves [11], so vegetation has been used in pesticide monitoring [12], [13] on the one hand, and on the other hand they has also been used to restore the contaminated sites [14], [15]. In fact, plants, as the sink for lipophilic pollutants due to their large surface area and associated lipids in plants [16], are also seriously affected with deleterious pollutants such as HCH. However, to our knowledge, although the residues difference of HCH isomers in different parts of plants was determined [5], [17], the phytotoxicity and toxic difference of HCH isomers in high plant is seldom considered. For the purpose of giving a comprehensive risk assessment on OCPs, studies of vegetation contamination caused by OCPs such as HCH and the mechanism of phytotoxicity are expected.

In the previous studies, the evaluation of phytotoxicity induced by organic chemicals focused on the basic physical indexes such as plant biomass, dry matter, and the rate of germination [18], [19]. Nevertheless, little was known about the phytotoxicity in plant photosynthetic and antioxidant defense systems. Although the changes of plant photosynthesis induced by OCPs were hardly referred to, many articles have reported that photosynthesis could be influenced dramatically when plants are subjected to a variety of adverse environmental conditions, such as chilling, desiccation and drought [20]–[22]. Meanwhile, excess generation of reactive oxygen species (ROS) can also be induced by those abiotic stresses; pesticide is also one of the adverse environmental factors that can induce oxidative stress [23], [24]. Considering ROS excess accumulation can lead to plant cell death, plant has evolved a whole antioxidant defense systems that can be divided into two categories: one that reacts with ROS and keeps them at low levels (peroxidase, superoxide dismutase and catalase), and one that regenerates the oxidized antioxidants (ascorbate peroxidase and glutathione reductase) [25]. Moreover, plant photosynthetic system and antioxidant defense system not only act independently of each other but also link intimately to perform signal function of ROS and response to adverse conditions. First, the sources of ROS are intimately correlated with photosystems in plants. It is generally accepted that PSI is the major site of superoxide generation in the photosynthetic electron transport chain, while superoxide (one kind of ROS) production by autoxidation of PSII components has been discussed. If superoxide forms in a short time and the antioxidant enzymes are unable to keep pace, superoxide as a signal molecule can trigger programmed cell death (PCD) in plants [26]. In addition, given that ROS scavenge by a number of enzymatic processes demands high energy, e. g. ascorbate peroxidase (APX) can detoxify H_2_O_2_ in the participation of NADPH [27]. Ott et al. [28] suggested that ROS would also be avoided in the first place by regulating photosynthetic electron transport.

In this study, the stereoselective toxicity of four HCH isomers absorbed from roots of *Arabidopsis thaliana* was studied. *Arabidopsis thaliana* is a common model plant widely used in studying plant physiology and toxicology. The changes of phenotype and substructure in *A. thaliana* treated by HCH isomers were observed. Parameters of photosynthetic system and enzymatic antioxidant defense system were first involved to investigate the stereoselective toxicity and oxidative stress when induced by HCH in model plant *A. thaliana.*

## Results

### The Effects of HCH Isomers on Morphology of *Arabidopsis thaliana*

**Figure 1:**
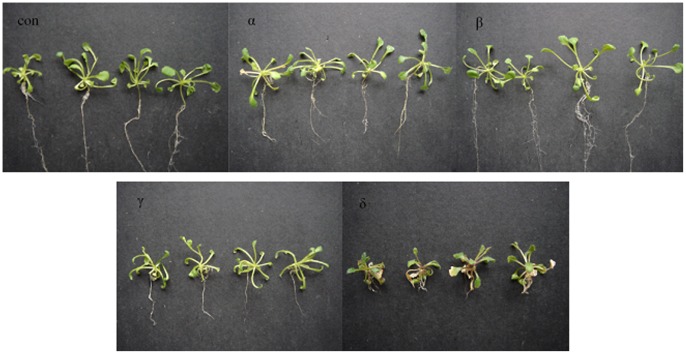
Phenotype of *Arabidopsis thaliana* exposed to α-, β-, γ- and δ-HCH (Exposure concentration: 5 mg/L) for four weeks.

As compared to controls, after treated by HCH isomers for four weeks, the pesticide affected the root dramatically, accessory roots numbers and the root length decreased. The root length of all isomers treated plants were inhibited obviously, and the order from long to short was con >β>α>γ>δ. At the macroscopic level, the leaf shape and size were not affected dramatically except for δ-HCH treatment, of which the leaves turned yellow. The fresh weight of the α-, β-, γ-HCH treatments were not significantly different when compared to controls, however, the fresh weight was dramatically decreased after four weeks exposure to δ-HCH isomer **(** **Figure 1** **,** **Table 1** **)**. Besides, the differences of root length and fresh weight among the four HCH isomers were also analyzed especially and shown in Table 1.

**Table 1:** Changes of root length and fresh weight in *Arabidopsis thaliana* exposed to α-, β-, γ- and δ-HCH for four weeks.

	con	α	β	γ	δ
**Root length (cm)**	10.2±1.0#	7.5±0.5^**a^	9.7±0.8^**b^	6.5±0.4^**c^	1.8±0.6^**d^
**Fresh weight (mg)**	26.0±3.2	25.0±3.6^a^	26.1±0.7^a^	30.3±2.9^a^	14.6±2.5* ^b^
**Root length inhibition (%)**	–	25.9	4.7	36.0	82.8

# mean value ± standard deviation;

* or ** indicate the values were significantly different as compared with controls (*p*<0.05 or *p*<0.01, respectively); Different letters show significant differences between values of the four HCH isomers (*p* = 0.05).

### Photos of Transmission Electron Microscopy of *A. thaliana* Leaves

**Figure 2:**
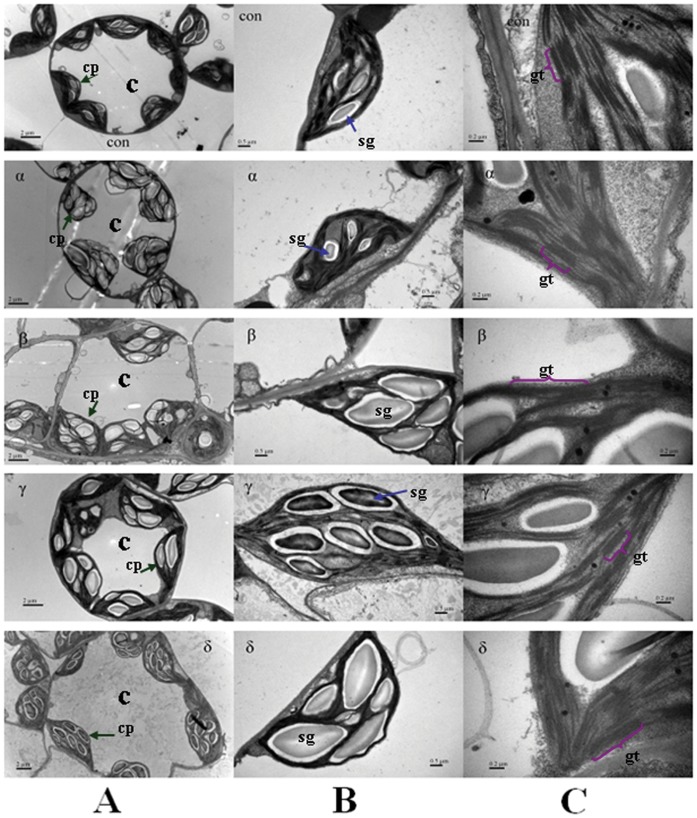
The substructure of *Arabidopsis thaliana* leaves treated by α-, β-, γ- and δ-HCH. (A) Mesophyll cell structure; (B) Chloroplast; and (C) Thylakoid structure. The Chloroplast (cp), starch granules (sg) and granal thylakoid (gt) are marked with colorful arrows respectively.

As shown in **Figure 2**, the substructure of *A. thaliana* leaves was employed to acquire the structure changes of chloroplast (cp) in where the photosynthesis takes place. Mesophyll cell was infected seriously by HCH (**Figure 2A**), the shape was not longer rounded or oval but irregular, especially for δ-HCH treatment. The number of chloroplasts in each cell was decreased. After exposing to HCH isomers, see **Figure 2B**, the morphology of chloroplast was also altered obviously. Expansion could be observed in β-, γ- and δ-HCH treatments, and the size of chloroplast was hardly affected in α-HCH treatment. The number and size of starch granules (sg) was increased in HCH treatments as compared with controls, δ-HCH treatment was the most affected one among the four isomers. For the structure changes of thylakoid, as shown in **Figure 2C**, the appressed regions of granal thylakoid (gt) were thinner since the extrusion by swelled starch granules and the stromal thylakoid links between the granal thylakoids were vague in HCH treatments. Furthermore, α-HCH treatment was the least affected one among the four isomers again.

### The Effects of HCH Isomers on Photosynthetic System of *Arabidopsis thaliana*

**Figure 3:**
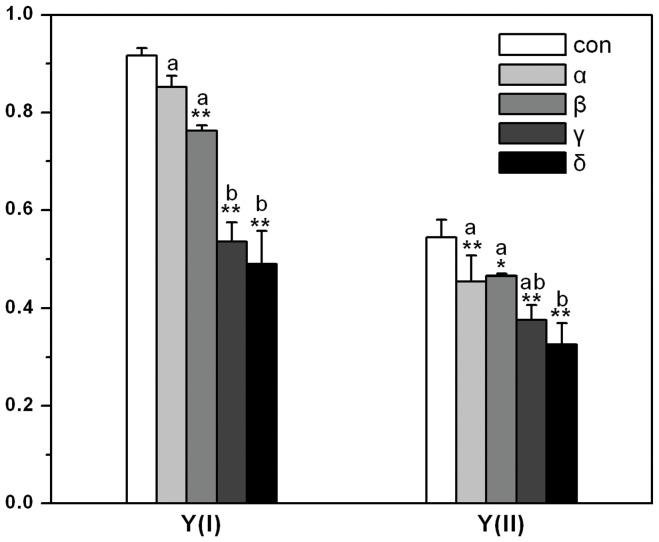
The effective quantum yield of photosystems [Y (I) and Y (II)] of *Arabidopsis thaliana* treated by HCH isomers. * and ** depict a statistically significant difference as compared to controls (*p*<0.05 or 0.01 respectively, ANOVA’s test). Different letters above the columns of HCH isomer treatments show statistically significant differences between them (*p* = 0.05).

In *A. thaliana*, the effective quantum yield was inhibited in both photosystem I and photosystem II (**Figure 3**). Compared to controls, statistical significance was shown almost in every treatment except for α-HCH treatment in photosystem I. The dramatic difference among the four isomers could also be observed and the highest and the least inhibition rate came from δ-HCH and α-HCH treatment, respectively.

**Figure 4:**
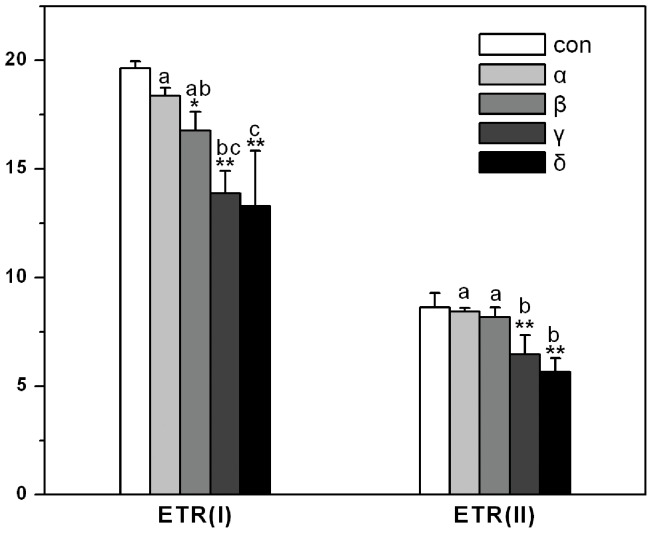
Changes of photosynthetic electron transport rates [ETR (I) and ETR (II)] in *Arabidopsis thaliana* treated by HCH isomers. Different letters above the columns of HCH isomer treatments show statistically significant differences between them (*p* = 0.05).

**Figure 5:**
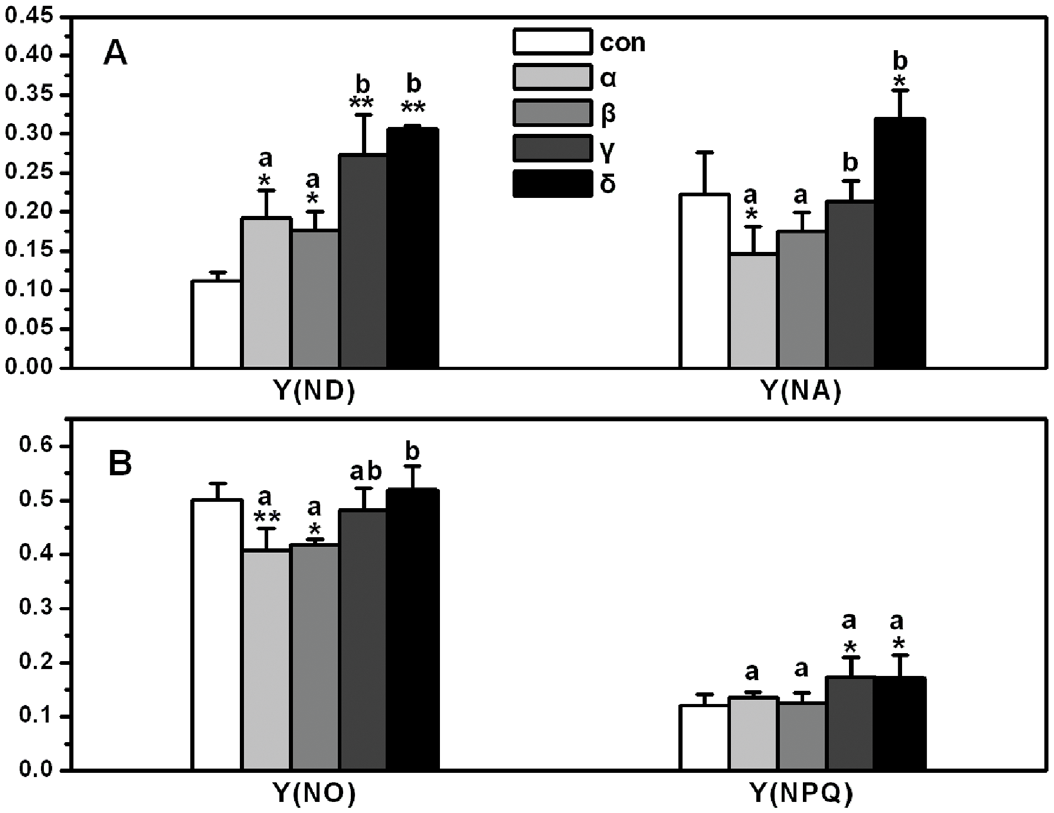
The quantum yields of non-photochemical energy dissipations in PSI and PSII of *Arabidopsis thaliana* treated by HCH isomers. (A), Y (ND) and Y (NA); (B), Y (NPQ) and Y (NO). Different letters above the columns of HCH isomer treatments show statistically significant differences between them (*p* = 0.05).

There were similar trend between photosynthetic electron transport rates (ETR) and the effective quantum yield of photosystems in *A. thaliana.* The inhibition effect of HCH isomers on ETR from serious to small was δ>γ>β>α in turn (See **Figure 4**). As shown in **Figure 5**, non-photochemical quantum yield of PSI caused by donor-side limitation [Y (ND)] was dramatically stimulated in all HCH treatments, especially in γ- and δ-HCH treatments. However, Non-photochemical quantum yield of PSI caused by acceptor-side limitation [Y (NA)] was inhibited in α-HCH treatment but stimulated in δ-HCH treatment. Quantum yields of non-light-induced non-photochemical fluorescence quenching [Y (NO)] were inhibited only in α- and β-HCH treatments, and Quantum yields of light-induced non-photochemical fluorescence quenching [Y (NPQ)] were stimulated in γ- and δ-HCH treatments. Moreover, the significant differences of all the photosynthetic parameters between the treatments of HCH isomers were also analyzed (Figure **3**-**Figure 5**).

### Activities of Anti-oxidative Enzymes and Malondialdehyde Content in *A. thaliana* Exposed to HCH Isomers

**Figure 6:**
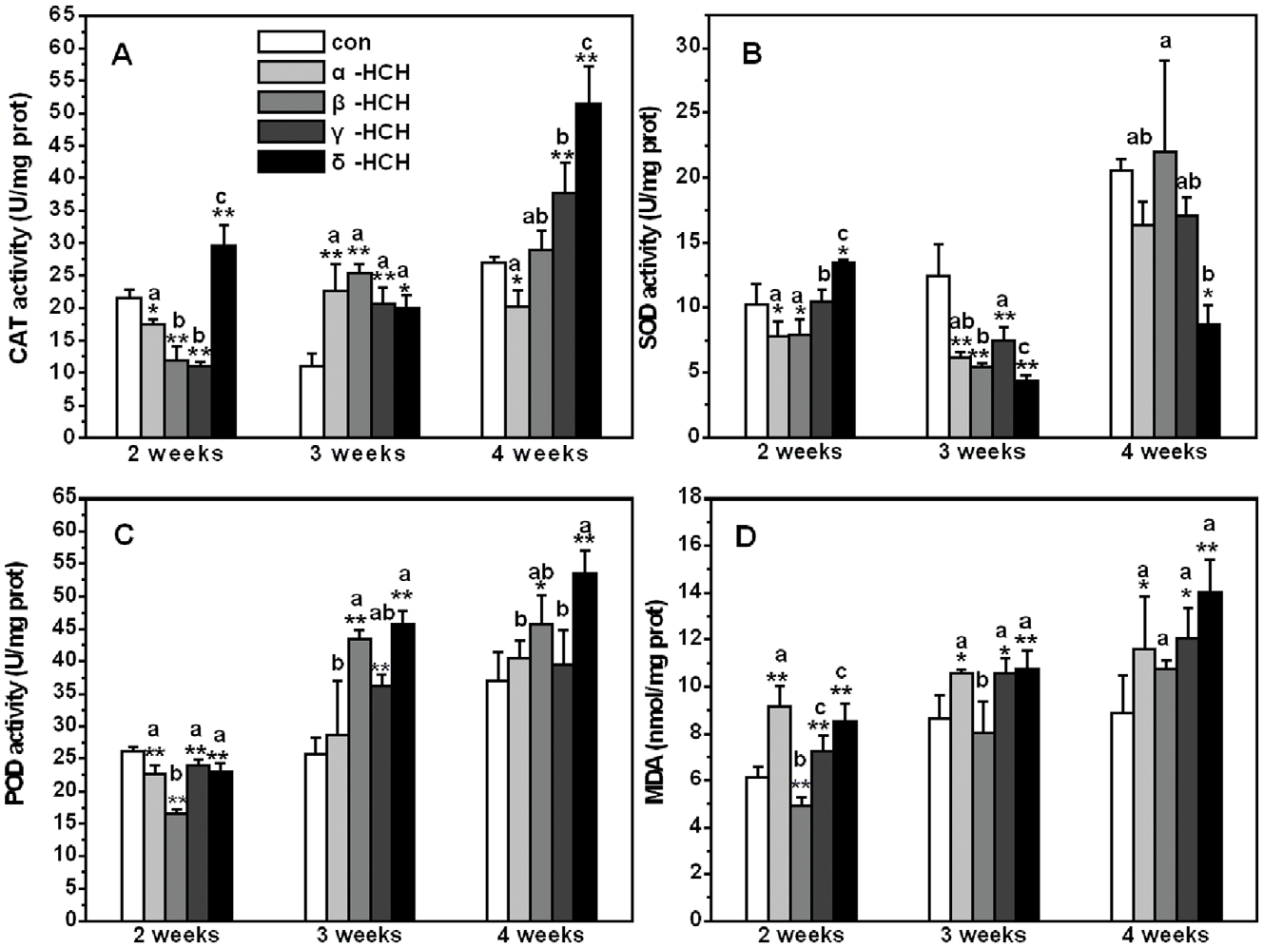
Activity of anti-oxidative enzymes and malondialdehyde content in *Arabidopsis thaliana* after different exposure time. (A) Activity of catalase (CAT); (B) Activity of superoxide dismutase (SOD); (C) Activity of peroxidase (POD); (D) Malondialdehyde (MDA) content.

The activity of the anti-oxidative enzyme was shown in **Figure 6A–6C**. Compared to controls, CAT activity was inhibited in the first two weeks after treatment with α-, β-, or γ-HCH treatments and was stimulated later on. However, the activity was always dramatically stimulated by δ-HCH. For the activity of SOD, inhibition was obviously observed in all HCH treatments at three weeks; however, after exposing for four weeks, the significant difference was only shown in δ-HCH treatment comparing with controls. The activity of POD was same as the CAT that a phenomenon of inhibition first and then stimulation was presented. POD activity was the most dramatically stimulated by δ-HCH and the least by α-HCH. The MDA level affected by the four HCH isomers was also investigated at different time. MDA content increased in all HCH treatments at different exposure time when compared to controls. Within the four isomers, δ-HCH treatment was also the most affected one and the β-HCH was the least affected one (**Figure 6D**). Similarly, the significant differences of all the anti-oxidative enzymes and MDA between the treatments of HCH isomers were specially analyzed (**Figure 6**).

## Discussion

Recently, phytotoxicity of HCH on nine plant species was conducted by Pereira et al. [19]. All of the species displayed signs under stress in response to the presence of HCH, although to different degrees. However, the reports about the toxic differences of HCH isomers in plant were rare despite there are lots of reports on stereospecific toxicity of HCH isomers in other organisms. For instance, Srivastavaa and Shivanandappa [29] investigated the toxicity of HCH isomers and its mechanism in Ehrlich Ascites tumor (EAT) cells. Their studies show differential cytotoxicity of α, β, γ, δ-HCH isomers, δ isomer being the most toxic and β the least. Nagata et al. [30] reported the differential effects of α, β, γ, and δ-HCH isomers on the GABA-induced chloride currents in three combinations subunits of GABA_A_ receptor expressed in human embryonic kidney cells. They found that differential actions of HCH isomers might produce variable effects on different regions of nervous systems and in different species of animals. Willett et al. [7] summarized the different toxicity of HCH isomers on animals: α-HCH resulted in the highest incidence of hepatic nodules and hepatocellular carcinomas in male mice orally exposed; γ-HCH has the lowest oral cancer potency factor as compared to other isomers in mice; Of the four isomers, β-HCH has the greatest physical and metabolic stability that reflected in the environmental and biological persistence of this isomer [31], [32]. Moreover, β-isomer could produce estrogen-like effects through non-classical estrogen-dependent mechanisms of action and therefore had the most important potential risks. After observing the phenotype and substructure of *Arabidopsis thaliana* exposed to HCH isomers, we concluded that the stereoselective toxic difference of HCH isomers was obvious, δ-HCH was the most toxic one while α-HCH the least. Therefore, the stereoselective toxic difference and action mode of HCH isomers in different organisms or even in different systems of same species was disparate. In our experiment, photosynthetic system and antioxidant defense system were first chosen to investigate the plant responses to HCH isomers. Photosynthesis is the basic course of organic chemicals synthesis by which plants and algae using sun energy. When plant treated with HCH, its photosynthetic system was down-regulated inevitably as we had discovered in results. In fact, although HCH toxicity mechanism on plants is unknown, over half the herbicides in current use act primarily on the light reaction of photosynthesis [33]. Oxidative stress is the situation by which many kinds of ROS were induced and the plant cells were damaged or killed because of the excessive accumulation of ROS. The antioxidant enzymes in plants would response to oxidative stress by up-regulating or down-regulating their activities. Pesticides such as paraquat could be one of the abiotic stresses that induced oxidative stress [34]. Both photosystems were affected dramatically by the HCH isomers in our study when we carried out our experiments on these two systems separately. Besides, as the main photosynthetic organelle, oxygen content of chloroplasts is higher, and the roles of ROS in plant responses to stress were undoubtedly focused on photosynthetic tissues such as cells with chloroplasts [35]. Moreover, as researchers have reviewed, photosynthetic cells indisputably have several unique ROS producing pathways and a multiplicity of ROS metabolizing systems under stress conditions [26]. And antioxidant enzymes in plants can scavenge the excessive accumulation of ROS that threaten the normal cell, therefore the antioxidant defense and photosynthetic systems are tightly interacted with each other when plant responses to stress.

There are a number of useful photosynthetic parameters that can be derived from saturation pulse-induced fluorescence analysis. The dual-PAM-100 measuring system has been used as an effective tool in determining these parameters that showed the effects of environmental stress on the plant photosynthesis [21], [22]. In this study, the effective quantum yield [Y (I) and Y (II)]) and photosynthetic electron transport rates [ETR (I) and ETR (II)] of PSI and PSII in *Arabidopsis thaliana* were profoundly decreased in all HCHs treatments especially in γ- and δ-HCH treatments. The energy absorbed by PSII is divided between the fraction used in photochemistry and that lost non-photochemically [36]. An increase in non-photochemical fluorescence quenching (NPQ) played as a protective process when Y (II) was decreased. **Figure 5B** depicted the changes of Y (NPQ) and Y (NO) that were protective indicator and injury index respectively in PSII. The increase of Y (NPQ) in γ- and δ-HCH treatments indicated that plant treated with HCHs could use its regulation mechanism such as a dissipation of energy in heat form to protect its photosynthetic system [20]. Similarly, Y (ND) is an important protective indicator in PSI when plants face to high light induced by environmental stress, if the value of Y (ND) is high, it shows that plant is suffered from excess light for one thing, and for another plant can protect itself by increasing heat dissipation. Because Calvin-Benson cycle is blocked, the electron in acceptor-side of PSI is accumulated. Then it leads to the increase of Y (NA), which is the mark of light injury [37], [38]. In current experiment, Y (ND) was higher in all the HCH treatments; however, the remarkable injury was only turned on in δ-HCH treatment, as shown in **Figure 5A**. Besides, some of the reactive oxygen species (ROS) such as triplet oxygen and superoxide generate in photosynthetic course [26]. Ott et al. [28] observed a decrease of electron transport rate between PSII and PSI and suggested that this regulation functions could limit superoxide formation under stress conditions. This is good news for plant how to avoid ROS toxicity but also restrict the efficiency of photosynthetic system as shown in **Figure 3**.

Growing evidence suggests a model for redox homeostasis in which antioxidant interaction acts as a metabolic interface for signals derived from metabolism and from the environment [39]. However, excessive ROS accumulation was harmful to plants [40]. Oxidative stress is a state of imbalance between generation of ROS like hydroxyl and superoxide radicals and the level of antioxidant defense system [41]. Since plant could not totally control the production and accumulation of ROS by electron transport course, oxidative stress occurred. The changes of activity of antioxidant enzymes and the increase of lipid peroxidation in our study confirmed that *Arabidopsis thaliana* plant, when exposed to HCHs isomers tried to eliminate the ROS by their enzymatic detoxification systems. Although little is known about the changes of antioxidant enzymes activity induced by HCHs in plants, there are also many reports about oxidative stress induced by HCHs in other organisms. For example, HCH induced oxidative stress in Ehrlich Ascites tumor cells was studied by Srivastava and Shivanandappa [42], their results could be characterized by glutathione depletion, lipid peroxidation (LPO), reactive oxygen species (ROS) production and inhibition of antioxidant enzymes, superoxide dismutase (SOD) and catalase (CAT). Moreover, effect of repeated oral administration of HCH on antioxidant defense system and lipid peroxidation in the rat testis was conducted by Samanta et al. [43], they also found that the pesticide elicited a significant decrease in the activities of cytosolic SOD and CAT.

Although the use of HCH has been inhibited, considering the long retention time of HCHs in environmental matrices and plant bioconcentration of HCHs [44], the pollution by HCHs in some special places was arresting, e. g. the surrounding areas of facilities that once used for HCH production. The environmental pollution by HCHs of high concentration [6] indicated that large residue of HCH isomers in special area was possible and deserved more concern. Plants, as one important part of ecosystem, an integral phytotoxicity study and residues detection are essential for understanding the global cycle and fate of OCPs, as well for assessing the risk of transfer to the trophic chain and for the development of phytoremediation techniques. Moreover, if we try to use vegetation to monitor or restore the environmental matrices contaminated by OCPs, more knowledge should be known, such as the tolerances of vegetation, transference and the metabolism of these pesticides after being absorbed by plant.

## Materials and Methods

### Regents and Plant Materials

The HCH isomers were purchased from Iprochem Co., Ltd (Shenzhen, Guangdong Province, China). *Arabidopsis thaliana* (ecotype Columbia [Col]) seeds were kindly provided by Prof. Jirong Wang (National Laboratory of Plant Molecular Genetics, Institute of Plant Physiology and Ecology, Chinese Academy of Sciences). All the organic reagents used in this experiment were analytically pure.

### Cultivation and Phenotype Analysis of *A. thaliana*

The *A. thaliana* seeds were sterilized with hypochlorite (4%) for 15 min first and with 75% ethanol for 1 min, repeated three times. After that, seeds were washed several times with distilled water and were vernalized at 4°C for 2–3 d. The MS medium was sterilized at 121°C for 20 min and 5 mg/L of α-, β-, γ- and δ-HCH was separately added in different conical beakers. The medium with different treatments was sufficiently blended and was removed into a 24-orifice plate respectively. All of the operations above were executed when the medium was still liquid. When the medium solidified, the vernalized seeds were sowed on. The plants used to taken morphological photos were sowed in vertical plates and all plates were sealed to avoid contamination. The plates were put into a constant temperature (25±0.5°C) culture room, equipped with cool-white fluorescence lights of 300 µmol/m^2^/s fluorescence intensity and a 12 h light/12 h dark cycle. After four weeks exposure, the plants were harvested for taking photos, meanwhile, the root length and fresh weight of the plants were measured.

### Substructure Detection by Transmission Electron Microscopy (TEM)

Leaf samples of controls and HCH-treated plantlets were sliced into crumbs of about 1 cm^2^square and fixed for over 2 h in cacodylate buffer solution containing 2.5% glutaraldehyde. Samples were then treated with 1.0% OsO_4_ for 1.5 h and dehydratedin acetone several times. After that, samples were embedded in epoxy resin. Ultra-thin sections (70–90 nm) were obtained using a Reichert Ultracutsultramicrotome, stained with uranyl acetate then by lead citrate. Finally, the samples of leaves were observed with a JEM-1230microscope (JEOL Ltd., Tokyo, Japan).

### Determination of Photosynthetic System Parameters in *Arabidopsis thaliana*

The photosynthetic system parameters in *Arabidopsis thaliana* Chloro fluorescence of PSII and PSI were measured concomitantly by using a Dual-PAM-100 fluorometer (Walz) connected to a computer. The automated induction and recovery curve routine in the Dual-PAM soft ware was used, with repetitive application of saturation pulses for the assessment of fluorescence and P700 parameters from which the quantum yields of PSI and PSII were derived by the software [45], [46]. Y (NO), Y (NPQ), Y (ND) and Y (NA) were calculated automatically by Dual-PAM soft ware and saved in a report file [47]. The ETR (I) and ETR (II) were also calculated by Dual-PAM software.

### Antioxidation Enzyme Extraction and Analysis

*A. thaliana* plantlets were grounded using a mortar with 2 ml PBS buffer (pH 7.4) on an ice bath. Every treatment was done with four replicates. After centrifuging at 2500 r/min for 10 min, the supernatant was collected to assay the antioxidant enzyme activity and malondialdehyde (MDA) level. The activity of superoxide dismutase (SOD), catalase (CAT), peroxidase (POD), and lipid peroxidation level reflected by MDA level was determined by using the kit came from Nanjing Jiancheng Bioengineering Institute (Nanjing, China). The related mechanisms of determination could refer to these articles [48]–[51].

### Statistical Analysis

Data were presented as mean±standard error of the mean (SD) and statistical significance was analyzed by Origin 6.0 (Microcal Software, Northampton, MA, USA). If the probability (*p*) was less than 0.05, the values of HCH treatments were considered significantly difference when compared to controls. For a statistical evaluation of difference between the four HCH isomers, the PASW Statistics 18.0 software (SPSS, Inc.) was used. Means of at least four repetitions of each parameter were assessed. The significance of the differences between every two treatments was evaluated by the analysis of the variance based on the method of Tukey contrasts (one-way ANOVA’ test) with a *p* value of 0.05.
